# Hyperparameter Tuning with High Performance Computing Machine Learning for Imbalanced Alzheimer’s Disease Data

**DOI:** 10.3390/app12136670

**Published:** 2022-07-01

**Authors:** Fan Zhang, Melissa Petersen, Leigh Johnson, James Hall, Sid E. O’Bryant

**Affiliations:** 1 Institute for Translational Research, University of North Texas Health Science Center, Fort Worth, TX 76107, USA; 2 Department of Family Medicine, University of North Texas Health Science Center, Fort Worth, TX 76107, USA; 3 Department of Pharmacology and Neuroscience, University of North Texas Health Science Center, Fort Worth, TX 76107, USA

**Keywords:** hyperparameter tuning, high-performance computing, machine learning, imbalanced data, mild cognitive impairment, Alzheimer’s disease

## Abstract

Accurate detection is still a challenge in machine learning (ML) for Alzheimer’s disease (AD). Class imbalance in imbalanced AD data is another big challenge for machine-learning algorithms working under the assumption that the data are evenly distributed within classes. Here, we present a hyperparameter tuning workflow with high-performance computing (HPC) for imbalanced data related to prevalent mild cognitive impairment (MCI) and AD in the Health and Aging Brain Study-Health Disparities (HABS-HD) project. We applied a single-node multicore parallel mode to hyperparameter tuning of gamma, cost, and class weight using a support vector machine (SVM) model with 10 times repeated fivefold cross-validation. We executed the hyperparameter tuning workflow with R’s *bigmemory*, *foreach*, and *doParallel* packages on Texas Advanced Computing Center (TACC)’s Lonestar6 system. The computational time was dramatically reduced by up to 98.2% for the high-performance SVM hyperparameter tuning model, and the performance of cross-validation was also improved (the positive predictive value and the negative predictive value at base rate 12% were, respectively, 16.42% and 92.72%). Our results show that a single-node multicore parallel structure and high-performance SVM hyperparameter tuning model can deliver efficient and fast computation and achieve outstanding agility, simplicity, and productivity for imbalanced data in AD applications.

## 1. Introduction

Over the last few years, machine learning (ML) has become an important research topic in the high-performance computing (HPC) community. HPC provides a large amount of opportunities, in terms of environments and resources, to help accelerate the process of ML. The ML community has also started to utilize the performance of HPC for better parallelization and scalability. For example, the number of articles on ML and HPC has reached 321,000,000 according to the search results from Google. However, applying ML and HPC to Alzheimer’s disease (AD) research is still relatively new. There was only 1 hit [1] from a PubMed search of ML, HPC, and AD (and 84 hits from Google), although the number of publications pertaining to ML and AD has greatly increased, from 294 in 2020 to 1582 in 2022.

Alzheimer’s is the most common cause of dementia, accounting for 60–80% of dementia cases. Its prevalence rate is about 11% among those aged 65 and older [2,3]. Collecting AD data while staying as close as possible to the prevalence rate in the population may create imbalanced data, which makes ML challenging [4]. For example, the Health and Aging Brain Study-Health Disparities (HABS-HD) data used here contained 1328 normal controls and 377 mild cognitive impairments (MCIs) and ADs [5–14]. In our preliminary analysis, all positive samples in the testing set were classified wrongly as normal, although our training set had almost 100% sensitivity and specificity. This was because the model learned from the imbalanced training set contained biases and made prediction too sensitive to the majority class, which consisted of normal controls for our HABS-HD data.

There are three ways to solve the imbalanced dataset problem: (1) downsampling, (2) upsampling, and (3) class weight optimization. Downsampling involves randomly removing observations from the majority class. Upsampling is the process of randomly duplicating observations from the minority class. However, downsampling will almost always lose information, while upsampling may lead to overestimation of the model performance and makes overfitting likely.

Hyperparameter tuning with class weight optimization has proven to be efficient in handling imbalanced data [15–17]. For example, John et al. worked with machine learning algorithms and imbalanced big data and found that, regardless of the classifier or encoding technique for categorical features, classifiers with tuned hyperparameters could yield better results than those with default values when classifying highly imbalanced big data [17]. Kong et al. compared hyperparameter optimization to default hyperparameters for both classification algorithms and resampling approaches and found that hyperparameter optimization could produce better results when classifying the imbalanced datasets [15]. Guido et al. presented a hyperparameter tuning method to improve model performance for imbalanced data [16].

HPC provides great opportunities to look into the imbalanced data problem while accelerating hyperparameter tuning efficiently [18,19]. In our previous work, we found that HPC could be used to significantly reduce computational time while maintaining the necessary accuracy for balanced AD data [1]. However, applying the workflow from [1] to imbalanced big data may cause an out-of-memory problem because the addition of a third parameter causes the memory usage to increase by x times, where x is the length of the class weight (normally, x is between 10 and 100). Loading the big imbalanced data chunk-by-chunk and then tuning it partially will not result in the same best parameters for all the chunks. Therefore, in this paper, we describe a hyperparameter tuning workflow with HPC and memory management for imbalanced data relating to prevalent mild cognitive impairment and Alzheimer’s disease in the HABS-HD project. We applied a single-node multicore parallel mode to hyperparameter tuning of gamma, cost, and class weight for a support vector machine (SVM) model with 10 times repeated fivefold cross-validation. We executed the hyperparameter tuning workflow with R’s *bigmemory*, *foreach*, and *doParallel* packages in Texas Advanced Computing Center (TACC)’s Lonestar6 system. The computational time was dramatically reduced by up to 98.2% for the high-performance SVM hyperparameter tuning model and the performance of cross-validation was also improved (the positive predictive value and the negative predictive value at base rate 12% were, respectively, 16.42% and 92.72%).

## 2. Materials and Methods

### 2.1. High-Performance Computing Structure

We used TACC’s Lonestar6 system for the hyperparameter tuning. Lonestar6 is the newest system in TACC’s Lonestar series of high-performance computing systems, which are deployed specifically to support Texas researchers. The system provides a balanced set of resources to support simulation, data analysis, visualization, and machine learning. Lonestar6 hosts 560 compute nodes with 5 TFlops of peak performance per node and 256 GB of DRAM (Table 1). The inter-node communication in Lonestar6 is supported by a Mellanox HDF Infiniband network, with capacities as high as 200 Gb/s.

### 2.2. R Pseudocode for Parallel SVM Hyperparameter Tuning

**Figure 1.**
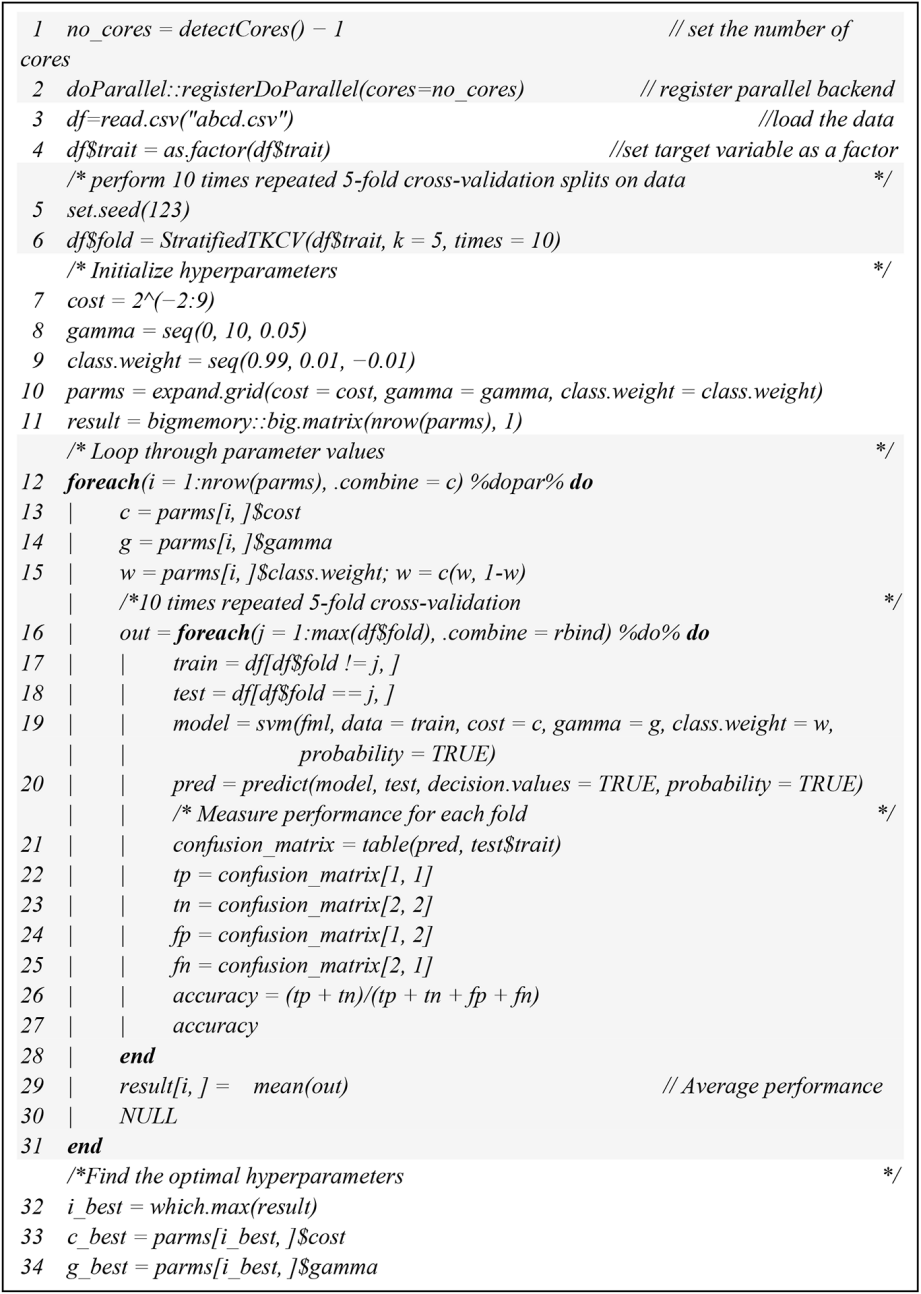
Pseudocode for my_script.R.

We submitted a single-node multicore parallel code to request 1 node (#SBATCH-N 1) with 128 tasks (#SBATCH-n 128). Before running *foreach()* in parallel (v1.5.2), we registered a parallel backend with *registerDoParallel()* in *doParallel()* (v1.0.17) (Figure 1). It is the easiest backend on most multicore systems. On Linux and Macintosh machines it uses fork system call, and on Windows machines it uses snow backend. It chooses automatically for the system.

Memory management is another important aspect in high-performance computing. On the one hand, embarrassingly, parallel problems require parallels solution because of the volume of data rather than the complexity of the algorithm. On the other hand, using more cores on a single node implies that each core has access to less memory. Using machine learning with high-performance computing can create problems when loading the data into RAM or the system may sometimes perform an operation for some data and then throw errors and stop working. To solve these problems, we used the *bigmemory* package (v4.5.36) to relieve the stress on system RAM and a combination of the *doParallel* (v1.0.17) and *foreach* (v1.5.2) packages for parallelized computation and, thus, faster computation (Figure 1).

### 2.3. 10 Times Repeated Fivefold Cross-Validation

We adopted 10 times repeated fivefold cross-validation [1] to reduce the noisy estimation of the optimal parameters of the ML model caused by a single run of the fivefold cross-validation [20]. Briefly, the fivefold cross-validation procedure where data samples are shuffled and stratified is repeated 10 times, and the mean performance across all folds from all runs is then used for the hyperparameter tuning.

### 2.4. Performance Measurement

In order to evaluate the performance of the hyperparameter tuning, we considered the following eight metrics:

(1) $$\text{Accuracy}=\frac{\text{TP}+\text{TN}}{\text{TP}+\text{TN}+\text{FP}+\text{FN}}$$

(2) Precision/PPV=TP/(TP+FP)

(3) Sensitivity=TP/(TP+FN)

(4) Specificity=TN/(TN+FP)

(5) NPV=TN/(TN+FN)

(6) $$\text{PPV12}=\frac{\text{Sensitivity}*12\text{\%}}{\text{Sensitivity}*12\text{\%}+(1-\text{Specificity})*(1-12\text{\%})}$$

(7) $$\text{NPV12}=\frac{\text{Specificity}*(1-12\text{\%})}{\text{Specificity}*(1-12\text{\%})+(1-\text{Sensitivity})*12\text{\%}}$$

and the area under the curve (AUC). Abbreviations: PPV, positive predicted value; NPV, negative predicted value; TP, true positive; FP, false positive; FN, false negative; TN, true negative; PPV12, positive predicted value at the base rate of 12%; NPV12, negative predicted value at the base rate of 12%.

## 3. Results

We downloaded the HABS-HD data from [10]; these data are available to the global scientific community to foster a more advanced understanding of the biological, social, cultural, and environmental factors associated with MCI and AD. The HABS-HD data used here contained 1328 normal controls and 377 MCIs and ADs (116 ADs and 261 MCIs). An imbalance occurred in this classification (class imbalance index = 0.31 according to the formula $I=K*\sum_{i=1}^{K}{\left(n_{i}-1/K\right)}^{2}$, where *K* is the number of classes, and *n*_*i*_ is the number of instances of class *i*) because the MCI and AD group had a very low proportion in the training data compared to the normal control group. Moreover, the MCI and AD group had significantly more males (*p* = 8.95 × 10^−7^ < 0.001) and significantly fewer years of education (*p* = 2.60 × 10^−7^ < 0.001) than the normal control group. There was no significant difference in age between the two groups (*p* = 0.002 > 0.001). Detailed demographic characteristics of the cohort were presented in [10].

The following seventeen blood marker variables were chosen to predict the status of prevalent MCI and AD: CRP, FABP3, IL_10, IL_6, Ab40, Ab42, Tau, NFL, PPY, sICAM_1, sVCAM_1, TNF_alpha, GLP_1, Glucagon, PYY, Insulin, and HOMA_IR (Table 2). Age, Gender, Hispanic (Hispanic or not), and Edu (years of education) were added as covariates [9,11,14,21].

**Figure 2.**
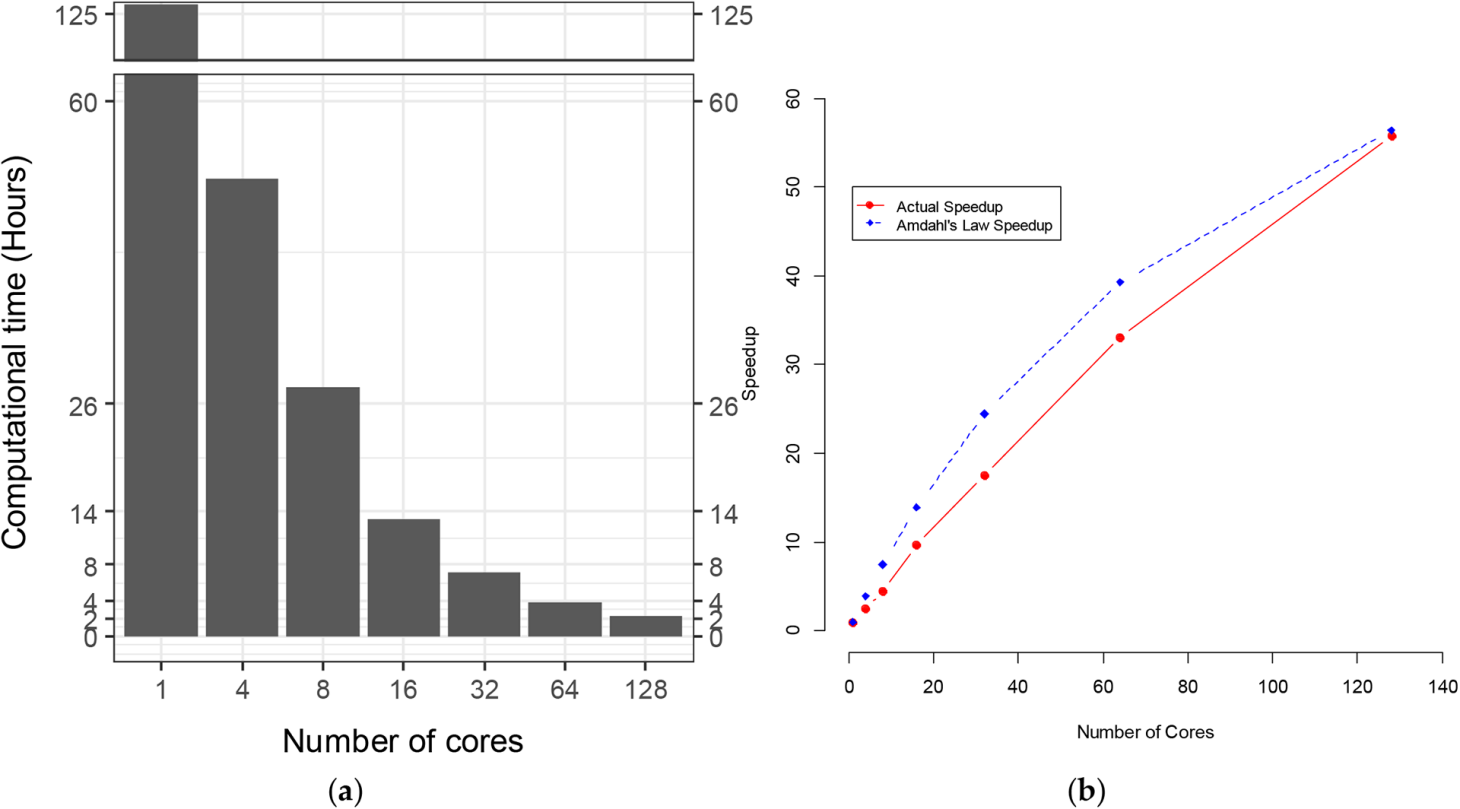
(**a**) Computational time vs. number of cores and (**b**) speedup vs. number of cores for imbalanced data.

We combined SLURM commands and R scripts to compare the computational time for hyperparameter tuning under different numbers of cores for a single node in Lonestar6 (Figure 1). The computational time for hyperparameter tuning for imbalanced data was inversely proportional to the number of cores for a single node (Figure 2a). For a single node, when we increased the number of cores from 1 to 128, the computational time was reduced but not linearly (Figure 2a). There was an overhead that reduced the efficiency, and not all of the tasks could be parallelized. The calculations of speedup vs. number of cores followed Amdahl’s Law at a parallel proportion of 99% (Figure 2b). The computational time initially spent for the hyperparameter tuning without using high-performance computing was 125.98 h. With 128 cores paralleled, the computational time decreased by up to 98.2% to 2.26 h. Measurement of the execution time for parallel SVM hyperparameter tuning was undertaken with the Sys.time() function in R (v4.1.2) by taking the difference between the times at the start and the end of the code chunk of the parallel SVM hyperparameter tuning.

We used the grid search method to find the optimal hyperparameters (gamma = 0.02, cost = 0.25, class.weight = [0.79, 0.21]). The boundaries for the two parameters, cost and gamma, were extended to [0.25, 512] and [0, 10], respectively, following the suggestion in [22], where the fine grid search had cost = [2, 32] and gamma = [2ˆ(−7), 2ˆ(−3)]. For the imbalanced data, we set the boundary for class.weight from 0.99 to 0.01 through a decrease of 0.01, which was equal to the class weight changing from [0.99, 0.01] to [0.01, 0.99].

For an imbalanced classification problem, the minority class is challenging to predict because there are few examples of this class. In our example, the minority class was the MCI and AD group and the majority class was the normal control group, which was about 3.5 times larger than the minority class (1328/377 = 3.52). Without tuning of the three hyperparameters, gamma, cost, and class.weight, the testing set in the 10 times repeated fivefold cross-validation failed to predict the characteristics of examples from the MCI and AD group with a sensitivity of 0 and specificity of 100% (Table 3). The positive predictive value and the negative predictive value at base rate 12% were, respectively, NaN% and 88.00%, which would definitely not be acceptable for clinical applications. After hyperparameter tuning, we successfully improved the differentiation of examples from the minority class (MCI and AD group) from the majority class (normal control group) with a sensitivity of 70.67% and specificity of 50.94% for the testing set in the 10 times repeated fivefold cross-validation (Table 4). The positive predictive value and the negative predictive value at base rate 12% were, respectively, 16.42% and 92.72%, which are acceptable for current clinical applications. Our results show that the hyperparameter tuning workflow with high-performance computing machine-learning for imbalanced Alzheimer’s disease data that we have presented can significantly reduce computational time while correcting imbalances.

We also used mlr.tuneParams in the mlr package [23] with parallel hyperparameter tuning in the Lonestar6 HPC system for comparison with our results. The grid search ranges for the three hyperparameters, cost, gamma, and class.weight, were the same. The 10 times repeated fivefold cross-validation was also performed. After 2.76 h running in a single-node 128 core parallel setup, the optimal hyperparameters were found at gamma = 0.02, cost = 32, and class.weight = [0.81, 0.19] and the final performance for the testing set exhibited the following values: sensitivity = 60.00%, specificity = 57.36%, PPV12 = 16.10%, and NPV12 = 91.32%). Compared to the paralleled mlr.tuneParams method, our hyperparameter tuning workflow could not only better adjust the imbalance bias with higher sensitivity, a higher positive predictive value and negative predictive value at base rate 12%, and slightly lower specificity, but also ran 18.1% faster.

## 4. Discussion

The HABS-HD project [9,14] collected data with unchanged prevalence of AD and MCI in the population. About 1 in 9 of those aged 65 and older (11%) in the United States has AD [3]. A study of a nationally representative sample of people aged > 65 years from the United States yielded a prevalence for MCI of approximately 12% to 18% [24]. The HABS-HD dataset contains 1328 normal controls and 377 MCIs and ADs (prevalence rate = 22.1% and class imbalance index = 0.31). A previous workflow [1] for hyperparameter tuning with HPC could have encountered an out-of-memory problem for the big imbalanced HABS-HD data. This study aimed to (1) solve the out-of-memory problem, (2) improve the model performance for imbalanced data, and (3) increase computation efficiency. We achieved the three goals by incorporating the *bigmemory*, *foreach*, and *doParallel* packages together with a single-node multicore parallel setup in the Lonestar6 HPC system. By switching to Lonestar6, we improved computation efficiency, which was up to four times faster than that in Talon3 [1]. Each compute node in Lonestar6 has two AMD EPYC 7763 64-core processors (Milan) and 256 GB of DDR4 memory. In contrast, each node in Talon3 has only two 2.4 GHz Intel Xeon E5–2680 v4 14-core processors and 64 GB memory. As an example, after loading into Lonestar6, the oasis longitudinal dataset from [1] only took 23.298 s and 12.119 s to complete for 28 cores and 128 cores, respectively, which was four times faster than Talon3 (which took 40 s).

### 4.1. Handling Imbalanced Data

Imbalanced data refer to outcome classes that appear with different frequencies. Such data pose a challenge for prediction since the default parameters of machine-learning algorithms are designed for balanced data. This can result in poor predictive performance, specifically for the minority class. For example, as shown in Table 2, in our binary classification problem for MCI and AD, the minority class (MCI and AD) appeared with a 22% probability. Applying a machine-learning algorithm naively without considering this class imbalance might lead to the algorithm always predicting the majority class (normal control), which here automatically resulted in 77.94% accuracy.

We performed and compared three different methods for handling the problem of imbalanced data for MCI and AD: (1) downsampling the normal control group, (2) upsampling the MCI and AD group, and (3) hyperparameter tuning. First, we randomly subsampled the majority class to reach the size of the minority class and obtained the following performance: sensitivity = 0.586, specificity = 0.528, PPV12 = 0.145, NPV12 = 0.903, and AUC = 0.616. Second, we randomly sampled from the minority class to reach the size of the majority class and achieved the following performance: sensitivity = 0.440, specificity = 0.660, PPV12 = 0.150, NPV12 = 0.896, and AUC = 0.576. The third method was the hyperparameter tuning method that we presented in the Results section, which outperformed the two resampling methods (downsampling and upsampling). This was consistent with the findings from [15]. Our results also demonstrated that the gamma, c, and class.weight values were key hyperparameters that could be used to train the most optimal SVM model using the RBF kernel for imbalanced data.

### 4.2. Memory Optimization

Running large datasets in parallel may result in the system running out of memory. The length of gamma was 201, the length of cost was 12, and the length of class.weight was 99. The total number of iterations for our hyperparameter tuning with 10 times repeated fivefold cross-validation was n(cost) × n(gamma) × n(class.weight) × 5 × 10, which was equal to 12 × 201 × 99 × 5 × 10 = 11,939,400. The size of the matrix for *foreach* to return was 11,939,400 × 10, which added up to 911 Mb RAM calculated by the object_size() function in the pryr package (v0.1.5). When monitoring our parallelization using the top command, the memory usage was too close to the Lonestar6′s memory ceiling, which is 256 G, especially when forking 128 cores. We applied the *bigmemory* package [25] to save memory. The *bigmemory* package is used to implement massive matrices and support their manipulation and exploration [25]. The data structures can be allocated to shared memory, allowing separate cores on the same node to share access to a single copy of the matrix [25]. In order to avoid R crashes, we used the *describe* and *attach.big.matrix* functions to access the shared memory. Further memory optimization and management was undertaken for the *foreach* expression. Retrieving values for *foreach* to combine with the *rbind* in a loop is known to be rather slow. The poor performance is caused by the need to repeatedly re-allocate memory for the growing data frame. Therefore we optimized the *foreach* expression by avoiding having *rbind* in a loop and returning a NULL value, which led to a greater performance gain (Figure 2).

### 4.3. Multinode Parallel

When using TACC’s Lonestar6, we could parallelize the jobs with multiple cores on a single node or multiple nodes. The computational times for the total of 128 cores on one, two, and three nodes were 2.265, 2.267, and 2.276 h, respectively. With the total number of cores unchanged, when the number of nodes was increased from 1 to 3, the computational performance actually decreased slightly. Distributing jobs on multiple nodes was slower than on a single node when the total number of cores remained unchanged. The loss in performance from a single node to multiple nodes may have been due to the expected switching from shared memory to inter-node transports.

Further, we performed experiments using all available cores in two and three nodes. The computational times were reduced to 2.226 h with 256 cores in two nodes (a reduction of 1.7% from 2.265 h with 128 cores in one node), and 2.204 h with 384 cores in three nodes (a reduction of 2.69% from 2.265 h with 128 cores in one node). The costs were 2, 4, and 6 SUs for 128 cores in one node, 256 cores in two nodes, and 384 cores in three nodes, respectively. We defined the performance as 1 divided by the computation time, and the performance cost ratio as the performance divided by the cost. The performance cost ratio was reduced from 0.2208 for 128 cores in one node by 49.12% to 0.1123 and by 65.74% to 0.0756 for 256 cores in two nodes and 384 cores in three nodes, respectively. Moving from a multinode to a single-node parallel setup, we could achieve a comparable computational time with nearly double the performance cost ratio.

## 5. Conclusions

The HABS-HD project collected unbalanced data from a real population. The data accurately represented the real population but posed challenges for machine learning with AD. We presented a hyperparameter tuning workflow with single-node multicore parallel high-performance computing and R’s *bigmemory*, *foreach*, and *doParallel* packages to improve prediction performance and computational efficiency for big imbalanced data relating to MCI and AD. Our results showed that the hyperparameter tuning workflow with HPC for big imbalanced data could correct the imbalance bias and reduced computational time by 98.2%, and it outperformed both the traditional downsampling and upsampling methods. Our results also showed that a single-node multicore parallel setup could achieve comparable computational time with a better performance cost ratio compared to a multinode parallel setup. The workflow can be applied in other fields with big imbalanced data that require accurate prediction and quick computation.

## Figures and Tables

**Table 1.** Node specifications for Lonestar6.

CPU	2× AMD EPYC 7763 64-Core Processor (“Milan”)
Total cores per node	128 cores on two sockets (64 cores/socket)
Clock rate	2.45 GHz (Boost up to 3.5 GHz)
RAM	256 GB (3200 MT/s) DDR4
Local storage	144 GB /tmp partition on a 288 GB SSD

**Table 2.** Description for the 17 variables.

Variable Name	Description	Average Value	Standard Deviation
CRP	C-reactive protein (CRP)	41,567,738	67,325,201
FABP3	Fatty acid-binding proteins (FABPs)	4953.23	2611.45
GLP_1	Glucagon-like peptide-1 (GLP-1)	1.07	2.09
Glucagon	Glucagon	49.46	49.98
IL_6	Human interleukin-6 (IL-6)	1.85	19.63
Insulin	Insulin	233.35	287.24
PPY	Pancreatic polypeptide (PPY)	400.87	491.56
PYY	Peptide YY (PYY), also known as peptide tyrosine tyrosine	35.46	38.33
sICAM_1	Intercellular adhesion molecule 1 (ICAM-1/CD54)	2,354,989	2,759,979
sVCAM_1	Vascular cell adhesion molecule 1 (VCAM-1/CD106)	3,681,600	4,361,946
TNF_alpha	Human tumor necrosis factor alpha (TNF-alpha)	3.57	15.14
Ab40	Aβ40 is a 40-amino acid proteolytic	244.08	78.38
Ab42	Aβ42 is a 42-amino acid proteolytic	11.7	3.71
Tau	Tau	2.38	1.17
NFL	Neurofilament light	18.47	14.08
IL_10	Human interleukin-10	0.42	0.64
HOMA_IR	Homeostatic model assessment for insulin resistance	1.9	2.92

**Table 3.** Performance for testing set in 10 times repeated fivefold cross-validation without hyperparameter tuning.

Actual		
Predicted	ADMCI	NC
ADMCI	0	0
NC	75	265
Precision/PPV	NaN%	
Accuracy	77.94%	
Sensitivity	0.00%	
Specificity	100.00%	
NPV	77.94%	
AUC	59.21%	
PPV12	NaN%	
NPV12	88.00%	

**Table 4.** Performance for testing set in 10 times repeated fivefold cross-validation after hyperparameter tuning.

Actual		
Predicted	ADMCI	NC
ADMCI	53	130
NC	22	135
Precision/PPV	28.96%	
Accuracy	55.29%	
Sensitivity	70.67%	
Specificity	50.94%	
NPV	85.99%	
AUC	64.73%	
PPV12	16.42%	
NPV12	92.72%	

## Data Availability

The datasets for this study can be found at https://apps.unthsc.edu/itr/ (accessed on 28 June 2022).

## References

[1] Zhang F; Petersen M; Johnson L; Hall J; O’Bryant SE Accelerating Hyperparameter Tuning in Machine Learning for Alzheimer’s Disease With High Performance Computing. Front Artif Intell 2021, 4, 798962. 34957393 10.3389/frai.2021.798962 PMC8692864

[2] Alzheimer’s Association. Alzheimer’s Disease Facts and Figures. Available online: https://www.alz.org/alzheimers-dementia/facts-figures (accessed on 21 March 2022).

[3] Hudomiet P; Hurd MD; Rohwedder S Dementia Prevalence in the United States in 2000 and 2012: Estimates Based on a Nationally Representative Study. J. Gerontol. B Psychol. Sci. Soc. Sci 2018, 73, S10–S19. 29669104 10.1093/geronb/gbx169 PMC6018928

[4] Iram S; Vialatte F-B; Qamar MI Chapter 1—Early Diagnosis of Neurodegenerative Diseases from Gait Discrimination to Neural Synchronization. In Applied Computing in Medicine and Health; Al-Jumeily D, Hussain A, Mallucci C, Oliver C, Eds.; Morgan Kaufmann: Boston, MA, USA, 2016; pp. 1–26.

[5] Hall JR; Johnson LA; Zhang F; Petersen M; Toga AW; Shi Y; Mason D; Rissman RA; Yaffe K; O’Bryant SE; Using Fractional Anisotropy Imaging to Detect Mild Cognitive Impairment and Alzheimer’s Disease among Mexican Americans and Non-Hispanic Whites: A HABLE Study. Dement. Geriatr. Cogn. Disord 2021, 50, 266–273. 34569492 10.1159/000518102 PMC8559764

[6] Hall JR; Wiechmann AR; Johnson LA; Edwards ML; O’Bryant SE Levels of alpha-2 Macroglobulin in cognitively normal Mexican- Americans with Subjective Cognitive Decline: A HABLE Study. Curr. Neurobiol 2019, 10, 22–25. 31061568 PMC6499402

[7] Johnson LA; Edwards M; Gamboa A; Hall J; Robinson M; O’Bryant SE Depression, inflammation, and memory loss among Mexican Americans: Analysis of the HABLE cohort. Int. Psychogeriatr 2017, 29, 1693–1699. 28629481 10.1017/S1041610217001016 PMC5647660

[8] King KS; Vintimilla RM; Braskie MN; Wei K; Hall JR; Borzage M; Johnson LA; Yaffe K; Toga AW; O’Bryant SE; Vascular risk profile and white matter hyperintensity volume among Mexican Americans and non-Hispanic Whites: The HABLE study. Alzheimer’s Dement. 2022, 14, e12263. 10.1002/dad2.12263 PMC8865739 35229016

[9] O’Bryant SE; Zhang F; Petersen M; Hall JR; Johnson LA; Yaffe K; Braskie M; Vig R; Toga AW; Rissman RA; Proteomic Profiles of Neurodegeneration Among Mexican Americans and Non-Hispanic Whites in the HABS-HD Study. J Alzheimer’s Dis. 2022, 86, 1243–1254. 35180110 10.3233/JAD-210543 PMC9376967

[10] O’Bryant SE; Johnson LA; Barber RC; Braskie MN; Christian B; Hall JR; Hazra N; King K; Kothapalli D; Large S; The Health & Aging Brain among Latino Elders (HABLE) study methods and participant characteristics. Alzheimer’s Dement. 2021, 13, e12202. 10.1002/dad2.12202 PMC8215806 34189247

[11] O’Bryant SE; Zhang F; Petersen M; Hall J; Johnson LA; Yaffe K; Braskie M; Rissman RA; Vig R; Toga AW; Neurodegeneration from the AT(N) framework is different among Mexican Americans compared to non-Hispanic Whites: A Health & Aging Brain among Latino Elders (HABLE) Study. Alzheimer’s Dement. 2022, 14, e12267. 10.1002/dad2.12267 PMC8828994 35155729

[12] Vintimilla R; Hall J; Johnson L; O’Bryant S The relationship of CRP and cognition in cognitively normal older Mexican Americans: A cross-sectional study of the HABLE cohort. Medicine 2019, 98, e15605. 31083252 10.1097/MD.0000000000015605 PMC6531144

[13] Vintimilla R; Reyes M; Johnson L; Hall J; O’Bryant S Cardiovascular risk factors in Mexico and the United States: A comparative cross-sectional study between the HABLE and MHAS participants. Gac. Med. Mex 2020, 156, 17–21. 32026882 10.24875/GMM.19005350 PMC8785358

[14] O’Bryant SE; Petersen M; Hall J; Johnson L; Team, H.-H.S Metabolic Factors Are Related to Brain Amyloid Among Mexican Americans: A HABS-HD Study. J. Alzheimer’s Dis 2022, 86, 1745–1750. 35253763 10.3233/JAD-215620 PMC9364418

[15] Kong J; Kowalczyk W; Nguyen DA; Bäck T; Menzel S Hyperparameter Optimisation for Improving Classification under Class Imbalance. In Proceedings of the 2019 IEEE Symposium Series on Computational Intelligence (SSCI), Xiamen, China, 6–9 December 2019; pp. 3072–3078.

[16] Guido R; Groccia MC; Conforti D A hyper-parameter tuning approach for cost-sensitive support vector machine classifiers. Soft Comput. 2022.

[17] Hancock J; Khoshgoftaar TM Impact of Hyperparameter Tuning in Classifying Highly Imbalanced Big Data. In Proceedings of the 2021 IEEE 22nd International Conference on Information Reuse and Integration for Data Science (IRI), Las Vegas, NV, USA, 10–12 August 2021; pp. 348–354.

[18] Liu Y; Li X; Chen X; Wang X; Li H; Ali R High-Performance Machine Learning for Large-Scale Data Classification considering Class Imbalance. J. Sci. Program 2020, 2020, 16.

[19] Guo J; Nomura A; Barton R; Zhang H; Matsuoka S Machine Learning Predictions for Underestimation of Job Runtime on HPC System; Springer: Cham, Switzerland, 2018; pp. 179–198.

[20] Zhang F; Petersen M; Johnson L; Hall J; O’Bryant SE Recursive Support Vector Machine Biomarker Selection for Alzheimer’s Disease. J Alzheimer’s Dis 2021, 79, 1691–1700. 33492292 10.3233/JAD-201254 PMC13270984

[21] O’Bryant S; Petersen M; Hall J; Johnson L; Yaffe K; Braskie M; Toga AW; Rissman RA; Rissman, for the HABLE study team. Characterizing plasma NfL in a community-dwelling multi-ethnic cohort: Results from the HABLE study. Alzheimers Dement 2022, 18, 240–250. 34310015 10.1002/alz.12404 PMC9228481

[22] Hsu C-W; Chang C-C; Lin C-J A Practical Guide to Support Vector Classification; National Taiwan University: Taipei, Taiwan, 2003.

[23] Bischl B; Lang M; Kotthoff L; Schiffner J; Richter J; Studerus E; Casalicchio G; Jones ZM mlr: Machine learning in R. JMLR 2016, 17, 5938–5942.

[24] Alzheimer’s Association. Mild Cognitive Impairment (MCI). Available online: https://www.alz.org/alzheimers-dementia/what-is-dementia/related_conditions/mild-cognitive-impairment (accessed on 16 June 2022).

[25] Kane M; Emerson JW; Weston S Scalable Strategies for Computing with Massive Data. J. Stat. Softw 2013, 55, 1–19.

